# Plant cooperation

**DOI:** 10.1093/aobpla/plv113

**Published:** 2015-09-25

**Authors:** Susan A. Dudley

**Affiliations:** Department of Biology, McMaster University, Hamilton, ON L8S 4K1, Canada

**Keywords:** Altruism, by-product mutualism, cooperation, facilitation, kin recognition, kin selection, multilevel selection, mutualism, reciprocation

## Abstract

The study of plant behaviour will be aided by conceptual approaches and terminology for cooperation, altruism and helping. The plant literature has a rich discussion of helping between species while the animal literature has an extensive and somewhat contentious discussion of within-species helping. Here, I identify and synthesize concepts, terminology and some practical methodology for speaking about helping in plant populations and measuring the costs and benefits. I use Lehmann and Keller's (2006) classification scheme for animal helping and McIntire and Fajardo's (2014) synthesis of facilitation to provide starting points for classifying the mechanisms of how and why organisms help each other. Contextual theory is discussed as a mechanism for understanding and measuring the fitness consequences of helping. I synthesize helping into four categories. The act of helping can be costly to the helper. If the helper gains indirect fitness by helping relatives but loses direct fitness, this is altruism, and it only occurs within species. Helpers can exchange costly help, which is called mutualism when between species, and reciprocation when within a species. The act of helping can directly benefit the helper as well as the recipient, either as an epiphenomenon resulting from behaviours under natural selection for other reasons, or because the helper is creating a mutual benefit, such as satiating predators or supporting a mutualism. Facilitation between species by stress amelioration, creation of novel ecosystems and habitat complexity often meets the definition of epiphenomenon helping. Within species, this kind of helping is called by-product mutualism. If the helping is under selection to create a mutual benefit shared by others, between species this is facilitation with service sharing or access to resources and within species, direct benefits by mutual benefits. These classifications provide a clear starting point for addressing the subject of helping behaviours.

## Abstract (precis)

Plants behave, but do plants help each other? This conceptual paper brings together theory developed for animal behaviour and insights from interspecific interactions between plants to address this question. The capabilities of plants and the empirical evidence indicate that altruism towards relatives, helping as a by-product of an adaptation to something else in the environment, and creating a mutual benefit are ways that plants may help other plants. Ongoing research indicates altruism towards relatives in competitive traits. Phenomena such as plant eavesdropping, pollinator attraction, predator swamping, and mutualisms with shared partners are potential examples of plant cooperation.

## Introduction

Plants not only behave, but plant behaviours are surprisingly complex. Plants can sense many aspects of the environment, from mechanical stimuli (Coutand 2010) to the presence of neighbours (Vandenbussche *et al*. 2005) to the identity of neighbours (Chen *et al*. 2012). Plants can express a conditional response to multiple aspects of the environment (Cahill *et al*. 2010). Though some plant behaviours involve foraging and competition (Cahill and McNickle 2011), others appear to benefit members of same species, including kin recognition in potentially competitive traits (reviewed in Dudley *et al*. 2013) and benefits from group associations (Harley and Bertness 1996; McIntire and Fajardo 2011). However, plant behaviour is a newly developing field. Should the theoretical basis of within-species plant helping behaviours be motivated by the large body of empirical literature from plants on between-species beneficial interactions, i.e., mutualisms (Leigh 2010) and facilitation (Brooker *et al*. 2008)? For example, researchers have used ‘intraspecific facilitation’ to refer to beneficial interactions within plant species (Harley and Bertness 1996; McIntire and Fajardo 2011). Or, should we bring the concepts of cooperation developed for animals into plant behaviour? Here, I bring together insights from mutualism and facilitation in plants with organizational frameworks from within-species cooperation and altruism theories developed for animals. I show that both fields share common themes and approaches to cooperation for plants.

## Naming Interactions Within and Between Species

The question of whether we should adopt the terminology from animal cooperation is not a simple one, since the terminology itself is a topic of considerable debate (Lehmann and Keller 2006; Bergmüller *et al*. 2007*b*; West *et al*. 2007; Forber and Smead 2015). Even the term ‘cooperation’ has a variety of definitions. The debate on terminology has roots in the varied theoretical approaches to positive interactions within and between species. Moreover, the debate is confounded by the varied ways in which the fitness consequences of positive interactions are assessed. For plants, the greatest controversy is whether plants can and do have mutually beneficial interactions within species. Consequently, plant researchers on positive interactions need a toolbox of terminology, theory and measurement of fitness consequences for empirical studies of within-species interactions.

**Figure 1.**
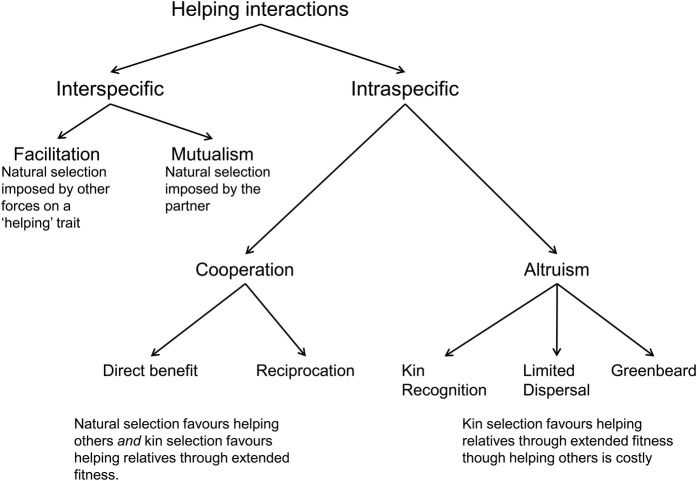
A consensus of the terminology of different mechanisms of helping, with expectations for how natural selection and kin selection are acting on these kinds of helping. Kin selection indicates indirect fitness benefits, and natural selection indicates direct fitness benefits.

Here, I primarily follow the conceptual framework developed by Lehmann and Keller (2006) for helping, cooperation and altruism based on a ‘direct fitness’ model (Fig. 1). The model estimates the ‘inclusive fitness’ of the focal individual or actor, the one providing the help. Inclusive fitness includes both the ‘direct fitness’ of the focal individual itself, and ‘indirect fitness’ resulting from helping a relative with shared genes. Increases in inclusive fitness may arise from the fitness benefits of helping, from reciprocation by a partner or from increases in indirect fitness resulting from helping a relative. This conceptual framework is particularly useful for considering the question of plant cooperation and altruism because it predicts fitness of the individual from the attributes of organisms and the features of their interactions.

Lehmann and Keller (2006) use ‘helping’ as the most inclusive term to describe any interaction within or between species where one partner increases another partner's fitness, i.e. provides a ‘benefit’. When one individual helps another of the same species, I will use ‘altruism’ when helping is costly to the helper, and ‘cooperation’ when helping directly benefits the helper (Lehmann and Keller 2006). For help between different species, I will use ‘mutualism’ for helping between species in different trophic levels where both benefit, and ‘facilitation’ for helping between species at the same trophic level, where at least one species benefits (Bronstein 2009; McIntire and Fajardo 2014).

Despite the debate on terminology (Lehmann and Keller 2006; Bergmüller *et al*. 2007*b*; West *et al*. 2007; Forber and Smead 2015), researchers tend to find the same broad categories for helping within species. Lehmann and Keller (2006) recognize three major divisions of helping within species: (i) ‘altruism’ or costly help, (ii) ‘cooperation’ which involves ‘reciprocation’ or exchange of costly help and (iii) ‘cooperation’ that involves ‘direct benefits’ for the helper, such that providing help is not costly (Fig. 1). While altruism can only evolve within species, other kinds of helping within species can share similar mechanisms with helping between species (Sachs 2006). However, the nature of natural selection becomes considerably more complex for helping within species, because of the potential for indirect fitness benefits through helping relatives. Indirect benefits provide the only mechanism by which altruism, i.e. costly helping, can evolve. As well, indirect benefits can increase the fitness benefits of cooperative behaviour, i.e. helping that increases the fitness of the helper.

## Multilevel Selection on Helping Traits

There are both conceptual and empirical reasons to use ‘multilevel selection’ to explore the fitness consequences of helping behaviours for the actor and the recipient. Multilevel selection is an extension of the phenotypic selection methodology (Lande and Arnold 1983). For phenotypic selection, the partial regression coefficients for fitness as a function of phenotypic traits, with traits and fitness measured on many individuals of the same generation, provide phenotypic selection gradients. For multilevel selection, these measures of traits and fitness are made in several groups to assess how group variation as well as individual variation in a trait affects individual fitness. Then, to measure the benefits of the helping trait to individuals within a group, the group traits, which are usually the group averages for a trait, are included in the regression to estimate the phenotypic selection gradients (partial regression coefficients) on group traits. This version of multilevel selection analysis is ‘contextual selection’ (Heisler and Damuth 1987; Goodnight 2005), and is complementary to ‘social selection’ (Wolf *et al*. 1999) as techniques for measuring how individual and group traits affect individual fitness (Goodnight 2015). Individual selection on a trait estimates the costs or benefits of the trait for the actor, while the group selection on the trait estimates the costs or benefits of a trait for others in the population. For simplicity, I will limit my discussion to contextual selection. Cooperation theory often discusses fitness consequences in terms of game theory scenarios between two partners **[see Supporting Information—File S1, Table S1 and Figure S1]** as a shorthand to describe how selection acts on traits where the outcome depends on the traits of the focal individual and the individual with which it interacts. However, contextual selection not only provides a description of how helping can be favoured, but also a methodology for measuring the natural selection on helping in plant populations.

**Figure 2.**
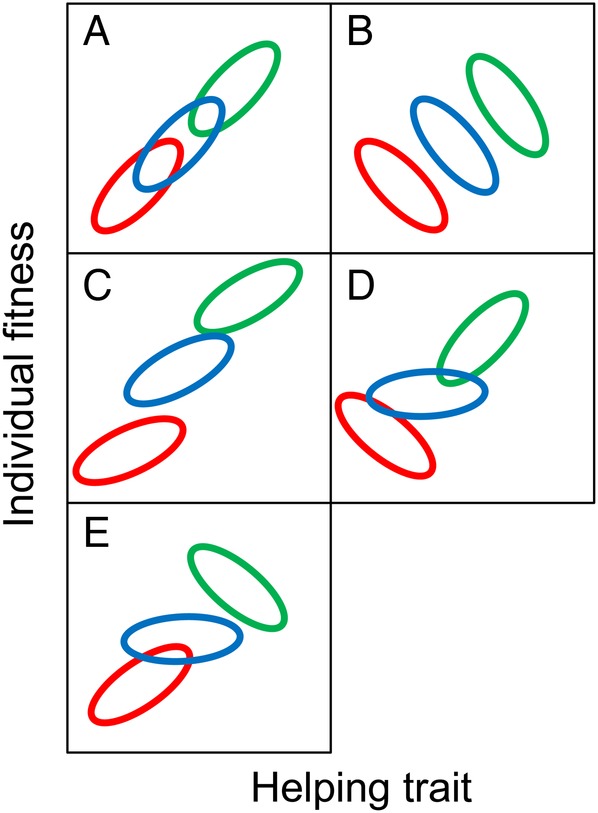
The expected relation between helping traits and fitness for different types of selection: (A) no group selection, (B) altruism, (C) synergism between group and individual selection, (D) negative frequency-dependent selection, (E) positive frequency-dependent selection. Ovals indicate clouds of observations from groups.

The fitness consequences of traits at the group level potentially ranges from very simple to quite complex. The simplest type of natural selection involves only individual selection, with no group selection. In the example (Fig. 2A), helping behaviour is positively associated with fitness without any effect of group membership. Contextual selection, which is based on partial regression coefficients, will determine that only individual level selection on helping is occurring (Heisler and Damuth 1987). In the other examples (Fig. 2B–E), positive group selection on a trait occurs, indicating that the higher group averages for the trait benefits others in the group, increasing their fitness (Heisler and Damuth 1987), regardless of the effect of individual selection. Any costs of the trait are measured in the individual selection component, i.e. the within-group relation of trait and fitness. In the case of altruism (Fig. 2B), helping is costly, so that individual selection favours reduced helping (Prisoners Dilemma game **[see Supporting Information—Table S2]**), while group level selection favours helping (Goodnight 2005). However, helping may also be beneficial for the individual. If, in addition to the group level selection, helping also benefits individual fitness in all groups (Harmony game **[see Supporting Information—Table S3]**), a synergistic pattern is created (Fig. 2C). If selection on helping is negatively frequency-dependent, then helping is only favoured when helping is rare (equivalent to the Snowdrift game **[see Supporting Information—Table S5]**), and not helping is favoured when others in the population do help (Fig. 2D). If selection on helping is positively frequency-dependent (equivalent to the Staghunt game **[see Supporting Information—Table S4]**), then helping is only favoured when helping is common (Fig. 2E).

## Mechanisms of Helping

Here, I discuss the three major divisions of helping within species recognized by Lehmann and Keller (2006); (i) altruism, (ii) cooperation which involves reciprocation and (iii) cooperation that involves direct benefits for the helper (Fig. 1). I identify the expected contextual selection for each type of helping. I relate cooperation within species to positive interactions between species. I also provide potential plant examples of these kinds of helping within species.

### Costly help directed towards relatives

Altruism (Figs 1 and 2B), can only evolve within a species, through providing costly help to relatives (Lehmann and Keller 2006). Helping relatives increases the actor's indirect fitness, as the relatives share the actor's genes. Consequently, an allele that favours costly helping of relatives can increase in the population, because the relatives are likely to have the same allele. The evolution of traits as a result of indirect fitness is known as kin selection. Hamilton's rule gives the conditions for altruism to evolve as *r*B > C, where *r* is the relatedness of the focal individual to the relatives it helps, B is the benefit of the behaviour to relatives and C is the cost of the behaviour to the focal individual (Hamilton 1963; Bourke 2014). The ability to direct help to relatives is crucial for kin selection (Lehmann and Keller 2006), either through local dispersal (also called high population viscosity), kin recognition or greenbeard effects (West *et al*. 2007). Even when helping provides direct benefits, directing that help to relatives adds indirect benefits, increasing the overall selection on the helping trait. Selection resulting from spatial structuring and group selection are essentially different theoretical approaches that measure the same processes as kin selection (Lehmann and Keller 2006; West *et al*. 2007) though see Goodnight (2015).

There is evidence for altruism and kin selection in plant functional traits related to competition. Plants have competitive behaviours (Novoplansky 2009; Cahill and McNickle 2011). Increases in competitive ability are selfish traits, as can be seen for the stem elongation response to neighbours. A more elongated and so taller plant in a dense stand both receives more light and shades its neighbours. Within a dense population, such elongated individuals have higher fitness (Dudley and Schmitt 1996). However, multilevel selection demonstrates that individuals in shorter or less elongated groups have higher fitness (reviewed in File *et al*. 2012*a*; Dudley *et al*. 2013). This pattern of multilevel selection, with opposing selection on group-level vs. individual traits (Fig. 2B), is supported by the outcome of artificial selection. In crop breeding, artificial selection for higher stand yield includes the development of dwarf cultivars that do not spend assimilate on excessive stem growth (Richards 2000). In a selection experiment imposing group and individual selection on plants in competition, individual selection for increased performance resulted in lower average group performance, but group selection for increased performance resulted in higher average group performance (Goodnight 1985). All these lines of evidence indicate that having a lower competitive ability is altruistic (Goodnight 2005), and so lowered competitive ability will only evolve through kin selection (Goodnight 2005; Lehmann and Keller 2006). More recent findings of kin recognition in plants (reviewed in Dudley *et al*. 2013) indicates that individuals can potentially direct help to relatives, as required for the evolution of altruism (Lehmann and Keller 2006). Traits implicated in competition, especially root allocation, show plasticity to the relatedness of neighbours (Dudley *et al*. 2013). However, more empirical work is needed to connect kin recognition responses with fitness under competition.

### Cooperation

**Figure 3.**
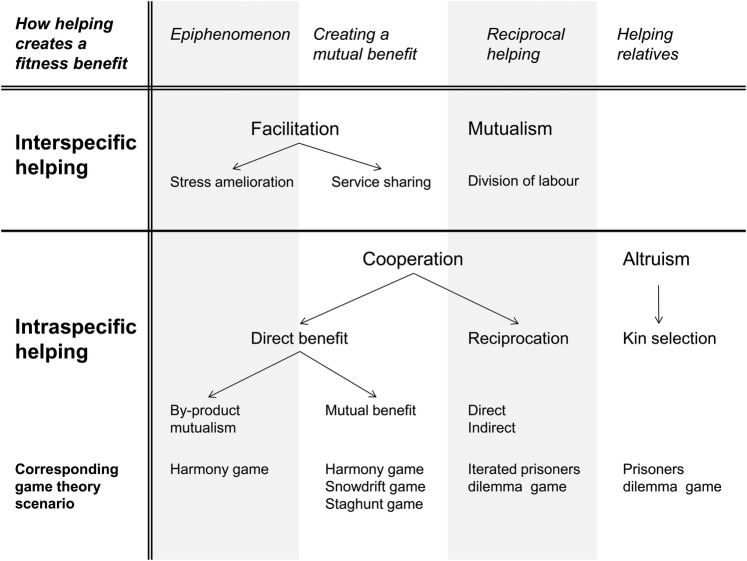
A mechanism-based classification of terminology for plant cooperation and altruism studies. This classification indicates shared mechanisms for within- and between-species helping, and identifies by-product helping and common benefit helping as different mechanisms. See Bergmüller *et al*. (2007*b*) for a discussion of direct and indirect reciprocity.

While altruism has no between-species analogue, cooperation within species is analogous to interactions between species (Fig. 3). Here, I first compare mutualism between species with reciprocation within species. I then compare facilitation between species with direct benefit cooperation within species, and argue for breaking up both processes into two separate mechanisms.

### Exchanges of help between and within species

When the partners are of different species (Fig. 3) and both trade help and benefit from their interaction, their interaction is called a mutualism (Bronstein 2009). Mutualisms are considered to arise from coevolution. Coevolution theory considers that each species affects phenotypic selection (Fig. 2A) on the helping traits of the other species (Clayton *et al*. 1999; Bronstein 2009). Mutualisms are well-known in plants, and include plant interactions with pollinators, symbiotic nitrogen-fixing bacteria and mycorrhizae (Leigh 2010). Mutualisms have an exchange of help between species, with division of labour (Leigh 2010).

When partners are of the same species (Fig. 3) and both trade help and benefit from their interaction, their interaction is called reciprocation (Lehmann and Keller 2006). Reciprocation has also been called reciprocal altruism or reciprocity. In reciprocation, the focal individual provides costly help to another individual of the same species, who in turn responds by providing costly help to the first individual. While each individual act of helping is costly, helpers ultimately gain increased fitness because of the benefits they receive from others they have helped. The Iterated Prisoner's Dilemma is the game theory **[see Supporting Information—File S1]** that corresponds to reciprocation. In quantitative genetics, recently developed models (Bijma 2014) that incorporate indirect genetic effects, i.e. genetic effects of individuals on the traits of other individuals in the population, offer an approach to understanding the evolution of reciprocation in multilevel selection. Non-human examples of reciprocity within species have been controversial in the animal literature (Bergmüller *et al*. 2007*a*; Raihani and Bshary 2011; André 2014; though see Dolivo and Taborsky 2015). The functional conditions (Lehmann and Keller 2006) that are required for reciprocation to increase fitness are repeated interactions and memory. These required conditions seem less possible for plants.

### Direct benefit help between and within species

When partners are of different species but come from the same trophic level (Fig. 3), and at least one partner benefits the other without incurring a cost, their interaction is called facilitation (McIntire and Fajardo 2014). Though definitions vary, generally an interaction is considered facilitation when the facilitated partner benefits, even if the facilitator providing the help gains no benefit or, in some definitions, is actually harmed (Bronstein 2009). Facilitation also occurs in animals, but it is recognized as a major force structuring communities in plants (McIntire and Fajardo 2014).

When partners are of the same species, the analogous interaction is direct benefit cooperation. Some plant researchers have called these interactions ‘within-species facilitation’ (Harley and Bertness 1996; McIntire and Fajardo 2011), but this creates additional terminology for the same processes, and so I suggest avoiding this usage. One partner receives a benefit from a single act of helping, and the other increases its fitness by helping, so there is no immediate or net cost to helping others.

One common aspect shared by facilitation (McIntire and Fajardo 2014) and direct benefit cooperation (Lehmann and Keller 2006) is how each comprises a multiplicity of mechanisms. McIntire and Fajardo (2014) and Leigh (2010) each provide a rigorous breakdown of several mechanisms of facilitation, using different paradigms. Lehmann and Keller (2006) and Connor (2010) include by-product mutualism and the Snowdrift game as mechanisms of direct benefit cooperation, but these are different mechanisms, with the first involving individual selection unrelated to helping others for the trait, while the second is negative frequency-dependent selection related to helping when the partner helps. Forber and Smead (2015) and Dugatkin (2002), in their discussions of direct benefit cooperation, focus on the Staghunt game, which represents positive frequency-dependent selection on helping when the partner helps. Here, I untangle these mechanisms, using the scheme of Leigh (2010) to divide helping with direct benefits into (i) helping as an epiphenomenon or by-product of other selection, and (ii) helping caused by sharing a common action or creating a mutual benefit without division of labour (Fig. 3).

In interspecific facilitation, helping is often an epiphenomenon or by-product (Fig. 3). In McIntire and Fajardo's (2014) classification of facilitation, mechanisms where one species facilitates another through habitat creation or amelioration of the stressful environment are likely epiphenomena. That is, the helping trait has evolved as a consequence of other agents of selection rather than as a result of natural selection arising from the species that is helped. A classic example of facilitation is the increased survival of cactus seedlings under nurse plants, which are adults of shrubs species whose proximity provides a favourable microclimate. Species differ in how much they help cactus seedlings. However, the effects of the plant canopy on the microclimate evolve in response to selection on how traits such as branching, leaf area index and leaf shape affect leaf temperature, photosynthesis and water loss. The cactus seedlings provide no known return benefit on the nurse plants, and so do not contribute to the natural selection on the nurse plants (Bronstein 2009; McIntire and Fajardo 2014).

For direct benefit cooperation within species (Lehmann and Keller 2006; Bergmüller *et al*. 2007*a*), the equivalent mechanism of epiphenomenon helping is ‘by-product mutualism’ (Fig. 3). Following the original definition by Brown (1983), by-product mutualism, sometimes called weak altruism, occurs when ‘clearly selfish’ behaviour helps others in the group (Eberhard 1975). Brown's (1983) definition of by-product mutualism indicates that natural selection always favours the helping trait regardless of what others do in the population. A mechanistic argument can be made for ‘plant eavesdropping’ (Karban *et al*. 2014) as a plausible example of epiphenomenon helping (by-product mutualism). Plants damaged by herbivores release volatile compounds that attract the predators of those herbivores. Other plants that sense (eavesdrop on) the volatiles up-regulate their own defences, increasing their fitness if they are attacked by herbivores (Karban *et al*. 2014). So the attacked plants are releasing volatiles to increase their own fitness, while the release of volatiles provides information that other plants can exploit.

Facilitation and direct benefit cooperation can occur through creating a mutual benefit or carrying out a joint action without division of labour (Leigh 2010; McIntire and Fajardo 2014). In McIntire and Fajardo's (2014) classification of mechanisms of facilitation, this kind of helping is called service sharing. Some facilitations and direct benefits involve a group who act together or create a common resource together, which can be a social good (Rankin *et al*. 2007). These mechanisms create benefits for the helper and the others in the population, such that they meet the criteria of direct benefits helping. Potential examples of service sharing and creating a mutual benefit include (i) mutually supporting a resource such as a mycorrhizal network, (ii) swamping predators by masting (producing seeds simultaneously) and (iii) creating a display of flowers that attracts and feeds pollinators. As Leigh (2010) specifies, these shared benefits do not involve specialization by the partners, unlike mutualisms between species at different trophic levels. Creating a mutual benefit can occur in intraspecific and interspecific interactions. However, these three mechanisms evolve through different mechanisms, depending on how the fitness consequences of creating the benefit depend on what others in the population do. Depending on the biology of the benefit, helping may only be favoured if others do not help, may only be favoured if others do help, or may always be favoured. The following examples suggest within-species natural selection for helping with direct benefits.

There are functional arguments to suggest that negative frequency-dependent selection (Fig. 2D) (Snowdrift game **[see Supporting Information—Table S5]**) is possible when multiple plant partners interact with one mycorrhizal network. When there is only a single plant and a single mycorrhizal fungus, the fungus and the plant are a straightforward mutualism (McNickle and Dybzinski 2013). The plant provides the fungus with carbohydrates that it requires for growth. In return, the fungus provides mineral nutrients that it acquires more efficiently than the plant does, and/or protection against pathogens (Powell *et al*. 2009). However, two or more plants attached to the same fungus are partners in supporting the fungus by donating carbohydrates to it (File *et al*. 2012*b*), thus creating a mutual benefit by supporting the mycorrhizae, which is a social good. It can be argued that when other plants do not support the mycorrhizae, a plant that does donate carbohydrates to a mycorrhizal fungus then gains resources it would not otherwise be able to access. But if donating carbohydrates is common in the population or community so that the mycorrhizae are well provisioned, then the best strategy for an individual is to freeload, and donate less carbohydrate while still getting a benefit (Rankin *et al*. 2007).

Evolution through positive frequency-dependent selection (Fig. 2E) (Staghunt game **[see Supporting Information—Table S4]**) is predicted when cooperation is rewarded only if the partner also cooperates. This reward structure indicates that, the more common cooperators are in the population, the more cooperation will be favoured by natural selection. One potential example of positive frequency-dependent selection in plants for a cooperative trait is masting as a mechanism of predator satiation. Masting, or mast fruiting, is a puzzling phenomenon where a population or community of long-lived species episodically produces large fruit crops, a so-called mast year. There are examples of synchrony in episodes of high production that are not driven by environmental variation, consistent with a biotic mechanism for synchrony (Kelly 1994; Curran and Leighton 2000; Schnurr *et al*. 2002). Predator satiation is one proposed agent of selection on synchronous reproduction, with so much fruit or seed produced that predators cannot eat it all. Both empirical evidence (Curran and Leighton 2000) and demographic modelling of masting and predation (Visser *et al*. 2011) indicate benefits from masting. The proximate mechanisms that would allow multiple individuals and even species to synchronize reproduction are currently the subject of research (Sanguinetti and Kitzberger 2008; Rossi *et al*. 2012; Miyazaki *et al*. 2014; Pearse *et al*. 2014).

Synergism (Fig. 2C) (Harmony game **[see Supporting Information—Table S3]**) with positive group and individual selection for height has been shown in the plant *Silene tatarica* (Aspi *et al*. 2003). The multilevel selection analysis demonstrated that an individual plant has increased fitness if it is tall. Moreover, an individual has increased fitness if it belongs to a tall subpopulation, regardless of its own height. Here, the authors speculate that height is involved in pollinator attraction, with taller groups and taller individuals being more visible to pollinators.

## Conclusions

I argue, therefore, for four mechanisms of help (Fig. 2), with three that apply to both within and between species types of helping. (i) Altruism within species: one individual provides costly help to another, increasing the other's fitness and reducing its own. (ii) Exchange of costly help: mutualisms between species and reciprocation within species involve partners that provide costly help in exchange for present or future costly help from another individual. (iii) Some facilitation between species, e.g. stress amelioration, and by-product mutualism within species can be the results of an epiphenomenon when the trait evolves in response to other agents of selection. (iv) Some facilitation between species, e.g. service sharing, and shared mutual benefit within a species can result from the creation of a mutual benefit or carrying out a joint action without division of labour. While all four mechanisms of help are likely important for plants, it is striking how much interesting plant life history falls into the fourth category of shared benefit or action. Even in animal cooperation, there are now calls for more research on direct benefit cooperation (Bergmüller *et al*. 2007*b*; Forber and Smead 2015).

This synthesis suggests several approaches to furthering research on plant cooperation and helping. The first is the assessment of the fitness consequences of putative helping plant traits for individuals and groups. Bringing together a mix of the common tools used in evolutionary biology, potentially including multilevel measurement of selection, adaptive arguments based on functional traits, modelling of evolutionary processes, selection experiments, comparison of populations and species, manipulation of traits and measurement of plasticity will be needed. The second is to use the functional approach of Lehmann and Keller (2006) to identify key abilities needed for different types of helping, and test to see if plants show them. The third, already in progress (McIntire and Fajardo 2011), is to look within species for same kinds of facilitation seen in interspecific interactions.

## Sources of Funding

Support for related research is provided by a Natural Sciences and Engineering Research Council of Canada Discovery Grant to S.A.D.

## Conflict of Interest Statement

None declared.

## Supporting Information

The following additional information is available in the online version of this article –

**File S1.** Game theory framework. Describes the game theory associated with different kinds of cooperation.

**Figure S1.** Cooperation games fall into four basic classes, depending on how the incentives to cooperate or defect for the focal player are affected by the decision of the second player. Redrawn from Helbing and Johansson (2010).

**Table S1.** Classic names motivated by the Prisoner's Dilemma game for the fitness of the focal player (row player), given the decisions of the focal individual and second player (column player) to cooperate or defect.

**Table S2.** This is an example of a fitness matrix that meets the assumptions of the Prisoner's Dilemma game.

**Table S3.** This is an example of a fitness matrix that meets the assumptions of the Harmony game.

**Table S4.** This is an example of a fitness matrix that meets the assumptions of the Staghunt game.

**Table S5.** This is an example of a fitness matrix that meets the assumptions of the Snowdrift game.

## References

[PLV113C1] André JB 2014 Mechanistic constraints and the unlikely evolution of reciprocal cooperation. Journal of Evolutionary Biology 27:784–795. 10.1111/jeb.12351 24618005

[PLV113C2] Aspi J, Jäkäläniemi A, Tuomi J, Siikamäki P 2003 Multilevel phenotypic selection on morphological characters in a metapopulation of *Silene tatarica*. Evolution 57:509–517. 10.1111/j.0014-3820.2003.tb01542.x 12703940

[PLV113C3] Bergmüller R, Russell AF, Johnstone RA, Bshary R 2007a On the further integration of cooperative breeding and cooperation theory. Behavioural Processes 76:170–181. 10.1016/j.beproc.2007.06.013 17719184

[PLV113C4] Bergmüller R, Johnstone RA, Russell AF, Bshary R 2007b Integrating cooperative breeding into theoretical concepts of cooperation. Behavioural Processes 76:61–72. 10.1016/j.beproc.2007.07.001 17703898

[PLV113C5] Bijma P 2014 The quantitative genetics of indirect genetic effects: a selective review of modelling issues. Heredity 112:61–69. 10.1038/hdy.2013.15 23512010 PMC3860160

[PLV113C6] Bourke AFG 2014 Hamilton's rule and the causes of social evolution. Philosophical Transactions of the Royal Society B: Biological Sciences 369 20130362. 10.1098/rstb.2013.0362 PMC3982664 24686934

[PLV113C7] Bronstein JL 2009 The evolution of facilitation and mutualism. Journal of Ecology 97:1160–1170. 10.1111/j.1365-2745.2009.01566.x

[PLV113C8] Brooker RW, Maestre FT, Callaway RM, Lortie CL, Cavieres LA, Kunstler G, Liancourt P, Tielbörger K, Travis JMJ, Anthelme F, Armas C, Coll L, Corcket E, Delzon S, Forey E, Kikvidze Z, Olofsson J, Pugnaire FI, Quiroz CL, Saccone P, Schiffers K, Seifan M, Touzard B, Michalet R 2008 Facilitation in plant communities: the past, the present, and the future. Journal of Ecology 96:18–34. 10.1111/j.1365-2745.2008.01373.x

[PLV113C9] Brown JL 1983 Cooperation—a biologist's dilemma. In: Rosenblatt JS, Hinde RA, Beer C, Busnel M-C, eds. Advances in the study of behavior, Vol. 13 New York: Academic Press, 1–37.

[PLV113C10] Cahill JF, McNickle GG 2011 The behavioral ecology of nutrient foraging by plants. Annual Review of Ecology, Evolution, and Systematics 42:289–311. 10.1146/annurev-ecolsys-102710-145006

[PLV113C11] Cahill JF Jr, McNickle GG, Haag JJ, Lamb EG, Nyanumba SM, St. Clair CC 2010 Plants integrate information about nutrients and neighbors. Science 328:1657 10.1126/science.1189736 20576883

[PLV113C12] Chen BJW, During HJ, Anten NPR 2012 Detect thy neighbor: identity recognition at the root level in plants. Plant Science 195:157–167. 22921010 10.1016/j.plantsci.2012.07.006

[PLV113C13] Clayton DH, Lee PLM, Tompkins DM, Brodie ED III 1999 Reciprocal natural selection on host-parasite phenotypes. The American Naturalist 154:261–270. 10.1086/303237 10506542

[PLV113C14] Connor RC 2010 Cooperation beyond the dyad: on simple models and a complex society. Philosophical Transactions of the Royal Society B: Biological Sciences 365:2687–2697. 10.1098/rstb.2010.0150 PMC2936175 20679112

[PLV113C15] Coutand C 2010 Mechanosensing and thigmomorphogenesis, a physiological and biomechanical point of view. Plant Science 179:168–182. 10.1016/j.plantsci.2010.05.001

[PLV113C16] Curran LM, Leighton M 2000 Vertebrate responses to spatiotemporal variation in seed production of mast-fruiting dipterocarpaceae. Ecological Monographs 70:101–128. 10.1890/0012-9615(2000)070[0101:VRTSVI]2.0.CO;2

[PLV113C17] Dolivo V, Taborsky M 2015 Norway rats reciprocate help according to the quality of help they received. Biology Letters 11 20140959 10.1098/rsbl.2014.0959 25716088 PMC4360107

[PLV113C18] Dudley SA, Schmitt J 1996 Testing the adaptive plasticity hypothesis: density-dependent selection on manipulated stem length in *Impatiens capensis*. The American Naturalist 147:445–465. 10.1086/285860

[PLV113C19] Dudley SA, Murphy GP, File AL 2013 Kin recognition and competition in plants. Functional Ecology 27:898–906. 10.1111/1365-2435.12121

[PLV113C20] Dugatkin LA 2002 Cooperation in animals: an evolutionary overview. Biology and Philosophy 17:459–476. 10.1023/A:1020573415343

[PLV113C21] Eberhard MJW 1975 The evolution of social behavior by kin selection. The Quarterly Review of Biology 50:1–33. 10.1086/408298

[PLV113C22] File AL, Murphy GP, Dudley SA 2012a Fitness consequences of plants growing with siblings: reconciling kin selection, niche partitioning and competitive ability. Proceedings of the Royal Society B: Biological Sciences 279:209–218. 10.1098/rspb.2011.1995 PMC3223689 22072602

[PLV113C23] File AL, Klironomos J, Maherali H, Dudley SA 2012b Plant kin recognition enhances abundance of symbiotic microbial partner. PLoS ONE 7:e45648 10.1371/journal.pone.0045648 23029158 PMC3460938

[PLV113C24] Forber P, Smead R 2015 Evolution and the classification of social behavior. Biology & Philosophy 30:405–421. 10.1007/s10539-015-9486-y

[PLV113C25] Goodnight CJ 1985 The influence of environmental variation on group and individual selection in a cress. Evolution 39:545–558. 10.2307/2408652 28561972

[PLV113C26] Goodnight CJ 2005 Multilevel selection: the evolution of cooperation in non-kin groups. Population Ecology 47:3–12. 10.1007/s10144-005-0207-2

[PLV113C27] Goodnight CJ 2015 Multilevel selection theory and evidence: a critique of Gardner, 2015. Journal of Evolutionary Biology 28:1734–1746. 26265012 10.1111/jeb.12685

[PLV113C28] Hamilton WD 1963 The evolution of altruistic behavior. The American Naturalist 97:354–356. 10.1086/497114

[PLV113C29] Harley CDG, Bertness MD 1996 Structural interdependence: an ecological consequence of morphological responses to crowding in marsh plants. Functional Ecology 10:654–661. 10.2307/2390176

[PLV113C30] Heisler IL, Damuth J 1987 A method for analyzing selection in hierarchically structured populations. The American Naturalist 130:582–602. 10.1086/284732

[PLV113C54] Helbing D, Johansson A 2010 Cooperation, norms, and revolutions: a unified game-theoretical approach. PLoS ONE 5:e12530. 20967256 10.1371/journal.pone.0012530 PMC2953489

[PLV113C31] Karban R, Yang LH, Edwards KF 2014 Volatile communication between plants that affects herbivory: a meta-analysis. Ecology Letters 17:44–52. 10.1111/ele.12205 24165497

[PLV113C32] Kelly D 1994 The evolutionary ecology of mast seeding. Trends in Ecology and Evolution 9:465–470. 10.1016/0169-5347(94)90310-7 21236924

[PLV113C33] Lande R, Arnold SJ 1983 The measurement of selection on correlated characters. Evolution 37:1210–1226. 10.2307/2408842 28556011

[PLV113C34] Lehmann L, Keller L 2006 The evolution of cooperation and altruism—a general framework and a classification of models. Journal of Evolutionary Biology 19:1365–1376. 10.1111/j.1420-9101.2006.01119.x 16910958

[PLV113C35] Leigh EG Jr 2010 The evolution of mutualism. Journal of Evolutionary Biology 23:2507–2528. 10.1111/j.1420-9101.2010.02114.x 20942825

[PLV113C36] McIntire EJB, Fajardo A 2011 Facilitation within species: a possible origin of group-selected superorganisms. The American Naturalist 178:88–97. 10.1086/660286 21670580

[PLV113C37] McIntire EJB, Fajardo A 2014 Facilitation as a ubiquitous driver of biodiversity. New Phytologist 201:403–416. 10.1111/nph.12478 24102266

[PLV113C38] McNickle GG, Dybzinski R 2013 Game theory and plant ecology. Ecology Letters 16:545–555. 10.1111/ele.12071 23316756

[PLV113C39] Miyazaki Y, Maruyama Y, Chiba Y, Kobayashi MJ, Joseph B, Shimizu KK, Mochida K, Hiura T, Kon H, Satake A 2014 Nitrogen as a key regulator of flowering in *Fagus crenata*: understanding the physiological mechanism of masting by gene expression analysis. Ecology Letters 17:1299–1309. 10.1111/ele.12338 25103959

[PLV113C40] Novoplansky A 2009 Picking battles wisely: plant behaviour under competition. Plant, Cell and Environment 32:726–741. 10.1111/j.1365-3040.2009.01979.x 19389051

[PLV113C41] Pearse IS, Koenig WD, Knops JMH 2014 Cues versus proximate drivers: testing the mechanism behind masting behavior. Oikos 123:179–184. 10.1111/j.1600-0706.2013.00608.x

[PLV113C42] Powell JR, Parrent JL, Hart MM, Klironomos JN, Rillig MC, Maherali H 2009 Phylogenetic trait conservatism and the evolution of functional trade-offs in arbuscular mycorrhizal fungi. Proceedings of the Royal Society B: Biological Sciences 276:4237–4245. 10.1098/rspb.2009.1015 PMC2821337 19740877

[PLV113C43] Raihani NJ, Bshary R 2011 Resolving the iterated prisoner's dilemma: theory and reality. Journal of Evolutionary Biology 24:1628–1639. 10.1111/j.1420-9101.2011.02307.x 21599777

[PLV113C44] Rankin DJ, Bargum K, Kokko H 2007 The tragedy of the commons in evolutionary biology. Trends in Ecology and Evolution 22:643–651. 10.1016/j.tree.2007.07.009 17981363

[PLV113C45] Richards RA 2000 Selectable traits to increase crop photosynthesis and yield of grain crops. Journal of Experimental Botany 51:447–458. 10.1093/jexbot/51.suppl_1.447 10938853

[PLV113C46] Rossi S, Morin H, Laprise D, Gionest F 2012 Testing masting mechanisms of boreal forest species at different stand densities. Oikos 121:665–674. 10.1111/j.1600-0706.2011.19953.x

[PLV113C47] Sachs JL 2006 Cooperation within and among species. Journal of Evolutionary Biology 19:1415–1418. 10.1111/j.1420-9101.2006.01152.x 16910971

[PLV113C48] Sanguinetti J, Kitzberger T 2008 Patterns and mechanisms of masting in the large-seeded southern hemisphere conifer *Araucaria araucana*. Austral Ecology 33:78–87. 10.1111/j.1442-9993.2007.01792.x

[PLV113C49] Schnurr JL, Ostfeld RS, Canham CD 2002 Direct and indirect effects of masting on rodent populations and tree seed survival. Oikos 96:402–410. 10.1034/j.1600-0706.2002.960302.x

[PLV113C50] Vandenbussche F, Pierik R, Millenaar FF, Voesenek LACJ, Van der Straeten D 2005 Reaching out of the shade. Current Opinion in Plant Biology 8:462–468. 10.1016/j.pbi.2005.07.007 16040269

[PLV113C51] Visser MD, Jongejans E, van Breugel M, Zuidema PA, Chen YY, Kassim AR, de Kroon H 2011 Strict mast fruiting for a tropical dipterocarp tree: a demographic cost-benefit analysis of delayed reproduction and seed predation. Journal of Ecology 99:1033–1044. 10.1111/j.1365-2745.2011.01825.x

[PLV113C52] West SA, Griffin AS, Gardner A 2007 Social semantics: altruism, cooperation, mutualism, strong reciprocity and group selection. Journal of Evolutionary Biology 20:415–432. 10.1111/j.1420-9101.2006.01258.x 17305808

[PLV113C53] Wolf JB, Brodie ED III, Moore AJ 1999 Interacting Phenotypes and the Evolutionary Process. II. Selection Resulting from Social Interactions. The American Naturalist 153:254–266. 10.1086/303168 29585974

